# Supplementary figures and images for: Investigating autism associated genes in C. elegans reveals candidates with a role in social behaviour
Source: PLoS One. 2021 May 27;16(5):e0243121. doi: 10.1371/journal.pone.0243121 (PMC8158995; doi:10.1371/journal.pone.0243121)

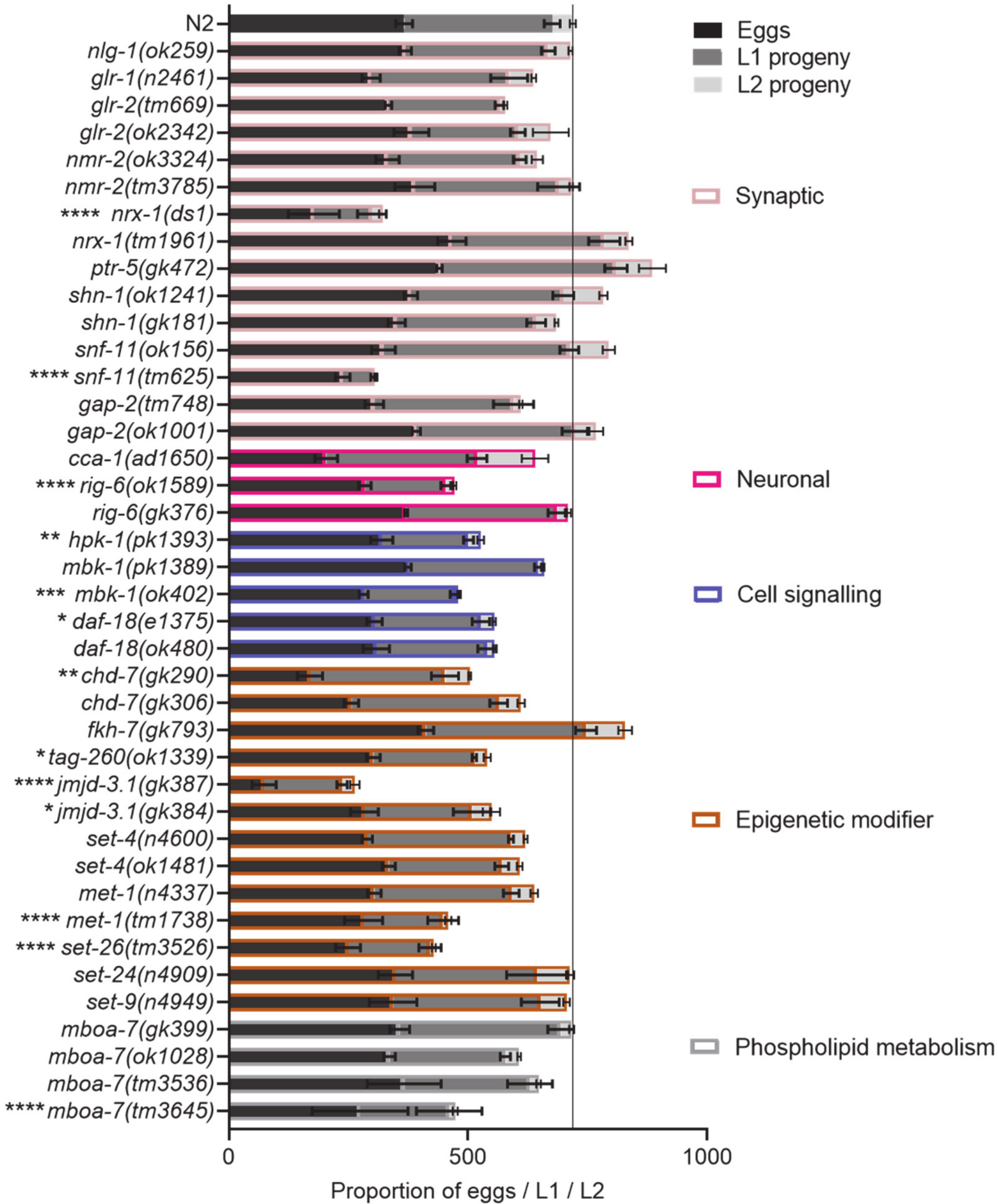

Supplement: S1 Fig — After a food leaving assay the number of eggs and progeny produced after 24 hours was counted. N2 and nlg-1(ok259) n = 19. All other mutants n = 3–4, where n refers to the number of replicates of an individual experiment. Strains were screened in batches across different days. Each batch consisted of 4–6 mutant strains and a paired wild-type control. Data plotted includes all wild-type controls. The black line indicates the total number of eggs and progeny produced by N2 control. All data shown as mean ±SEM. Statistical analysis performed using a two-way ANOVA and Dunnetts’s multiple comparison test; ns, p>0.05; *, p<0.05; **, p≤0.01; ***, p≤0.001; ****, p≤0.0001. All significance relates to the total number of eggs and progeny produced in comparison with N2 control. (TIF) [file pone.0243121.s001.tif]

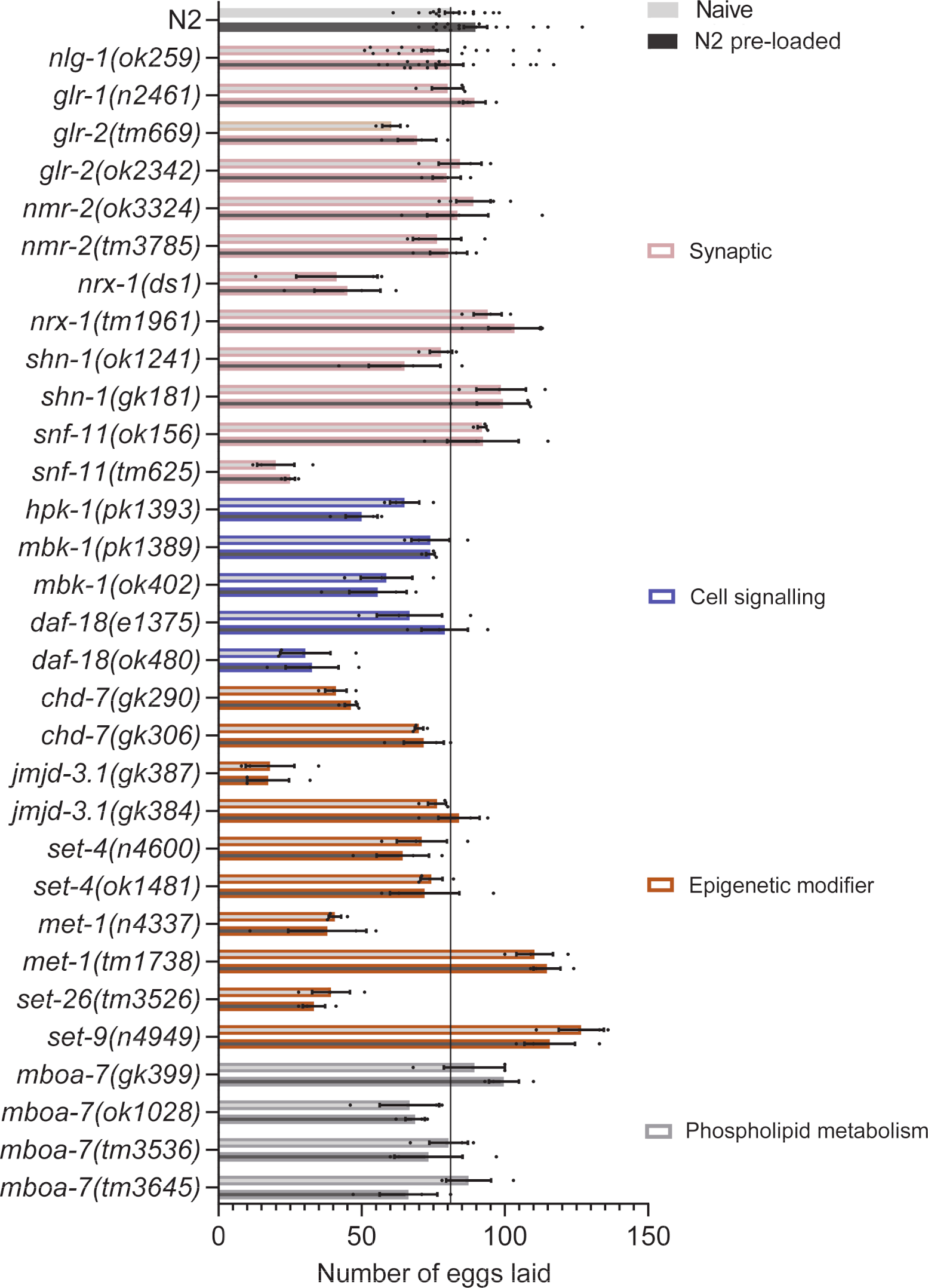

Supplement: S2 Fig — After a food leaving assay the number of eggs laid was quantified. The black line indicates the number of eggs laid by N2 control. All data shown as mean ±SEM. N2 and nlg-1(ok259) n = 16. All other mutants n = 3–4, where n refers to the number of replicates of an individual experiment. Strains were screened in batches across different days. Each batch consisted of 4–6 mutant strains and a paired wild-type control. Data plotted includes all wild-type controls. Statistical analysis performed using a two-way ANOVA and sidak’s multiple comparison test. No significance was identified when comparing eggs laid on a naïve and pre-loaded food lawn for each strain. (TIF) [file pone.0243121.s002.tif]
