# Supplementary material for: Investigating autism associated genes in C. elegans reveals candidates with a role in social behaviour
Source: PLoS One. 2021 May 27;16(5):e0243121. doi: 10.1371/journal.pone.0243121 (PMC8158995; doi:10.1371/journal.pone.0243121)
Supplement: Attachment — Submitted filename: Response to Reviewers.docx [file pone.0243121.s003.docx]

Dear Editor,

We are writing with regard to the manuscript #PONE-D-20-37216 titled ‘Investigating autism associated genes in C. elegans reveals candidates with a role in social behavior.’ We have taken the opportunity to submit a revised version of the manuscript and have addressed the points raised during the review process below. The reviewer comments are shown in italics and our responses are shown in blue.

Reviewer 1

*1) It seems to me that the food leaving assay may also be measuring the ability of the worms to move. If the animals have motility problems, then a great number of behavioral phenotypes are likely to be affected. The authors state they did not select strains that showed lethal, sterile or uncoordinated phenotypes, which is reasonable for the behavioural assays they wish to perform.*

We agree with the reviewer that the worms need to be able to move in order to leave the food. In the context of this work the dominant wild-type behaviour is dwelling on food and although motility is expressed during the worms short periods of roaming on food, using this as a proxy for motility will have clear limitations. We reinforce this view by drawing the referees attention to our published observations that the number of body bends a wild-type worm performs on food is low. In fact, in this work we show that this is the same for wild-type and food leaving deficient *nlg-1* mutants (Rawsthorne *et al.*, 2020). Consistent with this, preliminary analysis of some mutants investigated in this study shows very few body bends on food during quantification of motility for one minute per worm following a food leaving assay (Rebuttal Fig 1). As the reviewer highlights, we had already selected against such mutants with severe motility deficits during the bioinformatic filtering process prior to the candidate screen. Furthermore, during the observations we made on the selected mutants that emerged from our criteria based pipeline none exhibited a gross impairment in their ability to move.

We were mindful of the issue that a gross change in motility would make and selected the thrashing assay as a route to quantify core motility in an assay that is more sensitive to literature descriptions of the *unc* (uncoordinated) phenotypes (Buckingham and Sattelle, 2009; Koopman *et al.*, 2020; Frumkin *et al.*, 2014). To address one of the reviewers later comments (comment 6) we have performed a correlation between thrashing and food leaving. We identified a weak correlation between core motility and food leaving. Given the weak association we consider that the worms core motility has a minor contribution to the outcome of this context dependent assay and it is not the major determinant of the food leaving read out for the pipeline defined mutants defined in the earlier part of our study.

Rebuttal Fig 1: For referees consideration. N2 and mutant strain body bends motility on food. The number of body bends performed on food was quantified for one minute per worm at the end of the 30 minute period that worms were observed to define their food leaving. Analysis took place on naïve food lawns, containing no progeny, or N2 pre-loaded food lawns, containing approximately 150-200 *C. elegans* progeny. At least three separate experiments were performed for each strain, each paired to N2 control. Results show that the number of body bends performed on food is low for N2 control and is similar for all mutants tested.

*2) However, I feel that of their remaining candidate genes, they should have tested whether the mutants displayed uncoordinated phenotypes prior to using them for the food leaving assay.*

We hypothesise that the food leaving behaviour is the output of a complex interplay between several cues. Given the nature of how the assay is conducted the worms are exposed to interoceptive and exteroceptive cues. This includes gustatory, nutritional and mechanosensory inputs from the food. Superimposed upon this are progeny dependent cues that are precisely dosed in the assay by controlling the number of N2 progeny that are being used. The reviewer suggests testing mutants prior to the food leaving assay. In such an assay the interplay of sensory cues that are present within the food leaving assay would not be recapitulated. As mapped out above food impacts gross motility making it challenging to resolve subtle uncoordinated phenotypes.

We do however agree with the reviewer that the pattern of movement of the worm and the defining sub-behaviours is worthy of a high resolution investigation but this is beyond the scope of the detailing and throughput that the current submission is based on. Preliminary observations suggest that, in the presence of progeny, wild-type worms adapt their sub-behaviours particularly around the way in which they interact with the border of the food lawn. A detailed analysis of sub-behaviour in the food leaving assay is currently in progress and will attempt to define if single or multiple sub-behaviours can be ascribed to the various class of mutants mapped out in the current submission.

*3) Re: pharyngeal pumping
238. This shows that most mutants with reduced food leaving behaviour are capable of responding to food‐dependent sensory cues and co‐ordinating normal feeding behaviour.
Does the pharynx not continuously pump as it is largely an independent nervous system, thus I do not think this statement is accurate.*

The pharynx can continuously pump, however it’s activity (pump rate) is modulated in a context dependent manner. The rate of pumping can be modulated by food dependent signals, which can include extrapharyngeal 5-HT, dopamine and neuropeptides that act via volume transmission. Thus, the sustained pumping that these mutants show supports a largely intact food response from a complex circuit interaction. Our own published and unpublished observations detail more subtle modulation of the sensory modalities that have not been studied and are beyond the current submission. Thus we correct the text to read as

**Line 262 *‘This shows that most mutants with reduced food leaving behaviour are largely capable of responding to food‐dependent sensory cues and co‐ordinating normal feeding behaviour.’***

*4) 238. In addition, the cca‐1(ad1650) mutant which showed the most deficient pumping phenotype (Fig 3) did not show a food leaving phenotype (Fig 2), further suggesting that deficits in feeding behaviour are unlikely to explain differences in food leaving behaviour.
I do not think they can draw this conclusion from this observation.*

The *cca-1* mutant is a well-established mutant that has a deficient pumping phenotype (Steger *et al.*, 2005). In our hands we see the same deficit in pharyngeal pumping that has been previously published. *cca-1* does not have a food leaving phenotype. Taken together this supports the idea that supressed pharyngeal pumping is not a determinant of food leaving behaviour, since when it is suppressed in *cca-1* the food leaving behaviour is normal. Instead we consider that the major drive for the food leaving behaviour is the progeny dependent cue.

*5) 238. We plotted the relationship between the number of progeny produced by a mutant and the food leaving behaviour and showed that the two were correlated (Fig 5). Interestingly, this applies to the nrx‐1 and chd‐7 mutants for which the two alleles tested resulted in distinct social phenotypes (Fig 2). In each case the mutant that showed impaired social behaviour (Fig 2) also produced fewer progeny (S1 Fig). Producing fewer progeny means adult worms were exposed to fewer progeny‐derived social cues and could explain the low food leaving rate seen.

I really do not think they can draw these conclusions from these correlations. First, they have not measured (or identified) these progeny-derived social cues,*

We have measured the progeny-derived social cues in an original publication by identifying that they are largely produced by L2 stage worms, they produce a dose dependent cue and these cues are deficient in *daf-22* null progeny (Scott *et al.*, 2017). In absence of identified ascarosides we have used the precise dosing with 150-200 progeny to define the specific cue.

*6)`..and second these phenotypes could simply arise from overall impaired motility. In this aspect, it is essential to investigate general movement vs. food leaving behaviors.*

*I realize that the thrashing assay was conducted on these mutants, but I do not think this was done using automated measures (was this done manually?), and thrashing is an acute swimming response that does not fully recapitulate movement on plates during aging. Also, the authors should try to correlate the thrashing data to the food leaving phenotype, as they did for progeny vs. food leaving in Figure 5.*

The thrashing was performed manually and blind to the genotype, hence we consider this offers a high level of fidelity. Worms predominantly dwell during the 30 minute observation period of the assay, as evidenced by the very low rate of body bends, a readout for motility on solid agar (Rebuttal Fig 1). Hence, we consider that analysis of motility does not provide a robust readout for discriminating between mutant and wild-type motility in the food leaving assay. This is evidenced by our preliminary analysis of mutants *nlg-1(ok259), chd-7(gk306), chd-7(gk290)* and *set-4(ok1481)* which have impaired food leaving behaviour but cannot be discriminated from wild-type worms on the basis of body bends.

Thrashing behaviour has been shown to provide a readout for *unc* phenotypes (Buckingham and Sattelle, 2009; Koopman *et al.*, 2020; Frumkin *et al.*, 2014) and we consider it provides a readout for core motility to allow the identification of mutants with motility deficit.

We have correlated the food leaving behaviour with the thrashing phenotype and the R value suggests that there is a weak, positive linear correlation. Our interpretation of this is that the motility is not the major determinant. The correlation is now included as figure 7B and the figure legend has been appropriately updated.

*7) 263. Whilst some mutants showed minor disruption to thrashing behaviour (Fig 7) for most mutants thrashing did not predict food leaving behaviour. For example, nlg‐1(ok259) and set‐4 mutants showed deficits in food leaving behaviour (Fig 6) without any impairment to thrashing (Fig 7). Furthermore, four mutants of the mboa‐7 gene all showed impaired progeny‐induced food leaving behaviour with only one of these mutants having reduced thrashing (Fig 7).

I do not think this is accurate. Looking at the figure around 30% of the mutants showed thrashing rates less than N2. This section should be rewritten to reflect the reality of the phenotypes. Also, these data should be directly compared to each mutants corresponding food leaving phenotype.*

We have updated the text to read as follows:

**Line 343 *‘12 of the 31 mutants tested showed minor disruption to thrashing behaviour (Fig 7A). For the majority of mutants thrashing did not predict a food leaving phenotype. For example, nlg‐1(ok259) and set‐4 mutants showed deficits in food leaving behaviour (Fig 6) without any impairment to thrashing (Fig 7A). Furthermore, four mutants of the mboa‐7 gene all showed impaired progeny‐induced food leaving behaviour with only one of these mutants having reduced thrashing (Fig 7A). Consistent with these observations the correlation of thrashing behaviour with food leaving behaviour identified only a weak, positive linear correlation (Fig 7B). Taken together, this suggests that impaired motility may contribute but is not the major determinant of the food leaving behaviour.’***

*Perhaps I am reading this incorrectly, but there seems to be a huge discrepancy between the number of control worms and mutants tested for multiple experiments (figures 6,7, Supplementary figure 2 for example). One example is 88 N2 worms and 10-13 mutants in Fig. 7.*

The text in figure legends has been updated to clarify and read as follows:

***‘Strains were screened in batches across different days. Each batch consisted of X mutant strains and a paired wild-type control. Data plotted includes all wild-type controls.’***

‘X’ is replaced in the updated figure legends with the appropriate number for the experiment performed (see modified manuscript with track changes).

Reviewer #2:

*1. The methods were slightly unclear to me. In describing the Food Leaving Assay on p. 11, the authors mention animals were incubated on the plates for 24 hours (line 137) before assessing food leaving. Then in the next section (Pre-conditioned Food Leaving Assay), they mention that the plates were incubated for 2 hours (line 155) before leaving events were observed. I think this may be a typo, but if not, they need to justify the reason for the time differential in the plate incubation step.*

There are two parts to the screen, the first screen is performed by making observations at 24 hours, where gravid L4 plus 1 day old worms were cultivated for 24hrs, in which time they lay eggs and progeny mature to impact the original food lawn. Thus the assay of food leaving is done by measuring the now L4 plus 2 day old adults response to their own off-spring. We refer to this as a naïve incubation. The second screen is performed on pre-conditioned plates. In this assay N2 L4 plus 1 day old adults were placed onto a food leaving arena, allowed to lay 150-200 eggs and then removed. The eggs were then allowed to develop into progeny overnight. This largely recapitulates the levels of progeny that will be found at the 24 hour time point in the first screen. A new batch of 7 L4 plus one day old adult worms are then placed onto the assay plate, acclimatised for 2 hours before the food leaving was measured. This approach was first described in the Scott et al paper that is cited in the text.

To clarify, we have modified the text as follows:

**Line 139: *‘Plates were then incubated at 20^◦^C for 24 hours, during this time the seven assay worms lay eggs which produces a progeny laden lawn.’***

**Line 157*: ‘The plates were then incubated at 20◦C for 2 hours, this time course and pre-conditioning is equivalent to a 24 hour naïve incubation. Leaving events were then observed for 30 minutes as described above.’***

*2. At the start of the Results section, the authors observe that 84% of the class 1/2 ASD genes selected for this study have an orthologue in C. elegans. This appears to be a high rate of conservation of ASD genes between humans and C. elegans and is higher than the rate of conservation of genes in general or throughout these two genomes. Plus, a large proportion of the ASD risk genes appear to be essential genes causing lethality or sterility. This merits further comment as it fits with observations of conservation of risk genes in other neuropsychiatric disorders such as schizophrenia, which also has a strong neurodevelopmental component.*

Thank you for this insight we raise this point and have found reinforcing reference and highlight the overlap potential using the human data.

**Line 426: *We created a pipeline to prioritise human genes for investigation using C. elegans social behaviour. The pipeline identified 84% of the ASD genes ranked in categories 1 and 2 by SFARI have an orthologue in C. elegans. In general, the rate of conservation between the human and C. elegans genomes is 53% (Shaye and Greenwald, 2011) and suggests an enriched conservation of ASD genes between humans and C. elegans. A large proportion of the orthologous genes had known developmental phenotypes and were not selected for further analysis in this study. The analysis of such genes has the potential to provide broader insight into other neuropsychiatric disorders, such as schizophrenia given the overlap of risk genes between this disorder and ASD and the shared neurodevelopmental component of these two conditions. We selected 21 human genes for investigation using 40 C. elegans mutant orthologues.***

*3. The reference to ‘n’ in the Figure legends needs some further clarification. In some cases, e.g., Fig. 3, I think ‘n’ refers to the number of individual animals examined for those data. However, in other cases, e.g., Figs. 2, 4 & 5, ‘n’ appears to refer to the number of replicates of an individual experiment. If this interpretation is correct, it would help to explicitly state this to avoid confusion.*

Thank you, we have modified figure legends to clarify this.

*4, The authors did a commendable job of testing more than a single variant strain related to a particular gene, where possible. In many cases, there was concordance in the behavior of the different strains in the behavioral assays. There were a few notable exceptions that merit further discussion or exploration.*

*a) The nrx-1(ds1) allele suppressed food leaving whereas nrx-1(tm1961) did not. The corresponding SG1 strain has been outcrossed 3 times, whereas the FXO1961 strain has not been outcrossed.*

*So part of the difference between the two strains could be due to background gene variation. This could be explored or at least merits some discussion. RNAi against nrx-1 extends lifespan. Is the lifespan of either of these strains similarly affected? The nrx-1(tm1961) allele (truncated protein, presumably less functional) is associated with changes in enhanced slowing response (Rodriguez-Ramos et al. [2017] Behav Genet 47:596). Can this assay be used to probe the differences between these two strains? Finally, another strain VC1416 nrx-1(ok1649) is available potentially as a tiebreaker of sorts. These suggested experiments could help unravel what the true phenotype is for nrx-1 loss of function alleles. Further discussion of these issues is at least warranted.*

*b) The snf-11(tm625) allele suppressed food leaving whereas snf-11(ok156) did not. This situation is perhaps trickier to explain. Both are putative null alleles. RM2710 snf-11(ok156) has been outcrossed 6 times, mostly eliminating any stray variation, so this would be the standard for comparison, by most viewpoints. In this scenario, snf-11 would not be considered to affect social behavior in C. elegans. Background variation in snf-11(tm625), which has not been outcrossed, might then be responsible for the altered behavior. Mutant animals with the snf-11(ok156) allele have been reported to have some minor locomotor phenotypes such as increased sinusoidal amplitude during movement and an increase in forward velocity and roaming (Yemini et al. [2013] Nat Methods 10:877). Can these phenotypes be used to sort out the true role of snf-11 in food leaving behavior? In other words, can they determine whether spurious variants in FXO0625 snf-11(tm625) may cause the altered food leaving? Some of these issues and alternative interpretations need to be included in the Discussion.*

*Despite the comments in this last section, the effort to pin down contributions has been admirable. It would just help to show some of the limits to our ability to firmly assign cause and effect at this stage.*

Thankyou for highlighting this, we largely agree that analysis of multiple alleles for a single gene is reinforcing of the pattern of behaviour across the behavioural assays. We recognise that discrepancies in genetic disruption or variation in background and level of out crossing may underpin instances where there is not a concordance of food leaving behaviour between multiple alleles. Our future aim is to address this with a refined parameterization of the food leaving behaviour, which is ongoing and to combine this with precise genetic lesion of such genes to systematically test the alleles with equivalence of human mutations. We have updated the text to reflect the limitations and our future aim to address this.

We have modified the text in the discussion as follows:

**Line 449*: ‘******Where possible more than a single variant for a particular gene of interest was tested. Whilst in the majority of cases there was concordance in the behavioural output of variants, there were some exceptions and this may reflect differences in the number of times strains were outcrossed and potential differences in genetic backgrounds. Refined parametrization of the sub-behaviour that underpins the food leaving behaviour, combined with precise genetic lesion will facilitate the systematic testing of alleles with equivalence to human mutations.’***

Buckingham, S. D. and Sattelle, D. B. (2009) 'Fast, automated measurement of nematode swimming (thrashing) without morphometry', *BMC neuroscience,* 10, pp. 84-84.

Frumkin, A., Dror, S., Pokrzywa, W., Bar-Lavan, Y., Karady, I., Hoppe, T. and Ben-Zvi, A. (2014) 'Challenging muscle homeostasis uncovers novel chaperone interactions in Caenorhabditis elegans', *Frontiers in Molecular Biosciences,* 1(21).

Koopman, M., Peter, Q., Seinstra, R. I., Perni, M., Vendruscolo, M., Dobson, C. M., Knowles, T. P. J. and Nollen, E. A. A. (2020) 'Assessing motor-related phenotypes of Caenorhabditis elegans with the wide field-of-view nematode tracking platform', *Nature Protocols,* 15(6), pp. 2071-2106.

Rawsthorne, H., Calahorro, F., Feist, E., Holden-Dye, L., O’Connor, V. and Dillon, J. (2020) 'Neuroligin dependence of social behaviour in C. elegans provides a model to investigate an autism associated gene', *Human Molecular Genetics*.

Scott, E., Hudson, A., Feist, E., Calahorro, F., Dillon, J., de Freitas, R., Wand, M., Schoofs, L., O’Connor, V. and Holden-Dye, L. (2017) 'An oxytocin-dependent social interaction between larvae and adult C. elegans', *Scientific Reports,* 7(1), pp. 10122.

Shaye, D. D. and Greenwald, I. (2011) 'OrthoList: A Compendium of C. elegans Genes with Human Orthologs', *Plos One,* 6(5), pp. 12.

Steger, K. A., Shtonda, B. B., Thacker, C., Snutch, T. P. and Avery, L. (2005) 'The C. elegans T-type calcium channel CCA-1 boosts neuromuscular transmission', *Journal of Experimental Biology,* 208(11), pp. 2191-2203.
