# Supplementary material for: Investigating autism associated genes in C. elegans reveals candidates with a role in social behaviour
Source: PLoS One. 2021 May 27;16(5):e0243121. doi: 10.1371/journal.pone.0243121 (PMC8158995; doi:10.1371/journal.pone.0243121)
Supplement: Attachment — Submitted filename: Responses to Reviewers.docx [file pone.0243121.s004.docx]

Dear Editor,

We are writing with regard to the manuscript PONE-D-20-37216R1 titled ‘Investigating autism associated genes in C. elegans reveals candidates with a role in social behavior.’ We have taken the opportunity to submit a revised version of the manuscript and have addressed the points raised during the review process below. The reviewer/editor comments are shown in italics and our responses are shown in blue.

*Reviewer #1: In general the authors have done a good job addressing my concerns. There is one minor issue that I would like to be addressed before considering the manuscript for publication.
Line 343 ‘12 of the 31 mutants tested showed minor disruption to thrashing behaviour (Fig 7A). For the majority of mutants thrashing did not predict a food leaving phenotype. For example, nlg‐1(ok259) and set‐4 mutants showed deficits in food leaving behaviour (Fig 6) without any impairment to thrashing (Fig 7A). Furthermore, four mutants of the mboa‐7 gene all showed impaired progeny‐induced food leaving behaviour with only one of these mutants having reduced thrashing (Fig 7A). Consistent with these observations the correlation of thrashing behaviour with food leaving behaviour identified only a weak, positive linear correlation (Fig 7B). Taken together, this suggests that impaired motility may contribute but is not the major determinant of the food leaving behaviour.’
I am happy that the authors did take a second look at potential underlying motility phenotypes that could influence their assays. Afterall, many of these mutations likely affect development and thus it is not surprising that they may manifest phenotypes when assayed as adults. Eliminating animals with gross motility defects makes sense, but animals with less severe effects will likely confound other assays that require movement, namely most C. elegans behavioral assays.
The authors state that since there is a weak (a judgement call here) correlation which they interpret as motility defects not being the major determinant (another judgement call) of food leaving behavior. Instead I think it is more honest to simply state that they cannot discount general motility defects as a contributing factor, and they should say this explicitly.*

We have updated to text to read as follows:

**Line 353 ‘The bioinformatic pipeline filtered out mutant strains deficient in gross motility. In addition, we investigated the association between thrashing and food leaving behaviour and identified that there is a correlation between these behaviours (Fig 7B). This suggests that, whilst there are examples of mutants that have impaired food leaving in the absence of a thrashing phenotype, we cannot discount more subtle motility defects contributing to the summed food leaving response.’**

*Journal Requirements:*

*Please review your reference list to ensure that it is complete and correct. If you have cited papers that have been retracted, please include the rationale for doing so in the manuscript text, or remove these references and replace them with relevant current references. Any changes to the reference list should be mentioned in the rebuttal letter that accompanies your revised manuscript. If you need to cite a retracted article, indicate the article’s retracted status in the References list and also include a citation and full reference for the retraction notice.*

Thank you for bringing this to our attention. The article in question has not been retracted but a correction and corrigendum have been made to the original publication, this information has now been included in the reference list as follows:

**Line 646 ‘Chalasani SH, Chronis N, Tsunozaki M, Gray JM, Ramot D, Goodman MB, et al. Correction: Corrigendum: Dissecting a circuit for olfactory behaviour in Caenorhabditis elegans Nature. 2016;533(7601):130. doi: 10.1038/nature16515.’**

The corrections made to this article do not impact the data or conclusions made regarding the defective local search behaviour of glr-1(n2461) which is cited in table 1 of this manuscript.
